# Diabetic Kidney Disease and COVID-19: The Crash of Two Pandemics

**DOI:** 10.3389/fmed.2020.00199

**Published:** 2020-05-06

**Authors:** Luis D'Marco, Maria Jesús Puchades, Maria Romero-Parra, Jose Luis Gorriz

**Affiliations:** ^1^Hospital Clínico Universitario, Institute of Health Research (INCLIVA), Valencia, Spain; ^2^Universidad de Valencia, Valencia, Spain

**Keywords:** Covid-19, diabetic kidney disease, chronic kidney disease, cardiovascular disease, renal damage

The prevalence of diabetes mellitus (DM) is around 425 million people worldwide. Thus, the predictions for 2,045 is that will grow to over 600 million (1). Diabetic kidney disease (DKD) is a major cause of morbidity and mortality in diabetes and estimations report that ~30–40% of DM patients will develop DKD. In this regard, chronic kidney disease (CKD) is associated with most of the excess of all-cause and cardiovascular mortality in patients with diabetes. DM-affected patients are prone to infections due to immune dysfunctions (2). Moreover, DM patients with DKD express a chronic systemic inflammation that contributes to the immunosuppressed state that accounts for infectious complications, which together determine the morbidity and the mortality that is associated with these patients.

Due to how quickly it has spread, severe acute respiratory syndrome coronavirus 2 (SARS-CoV-2), the virus that causes coronavirus disease in 2019 (COVID-19) pandemic, will probably emerge as one of the most relevant infectious diseases of this century. Although governments everywhere plan for pandemics because their impact can cause sharp shocks to economies and societies, COVID-19 represents a real challenge and will require a substantial surge in health systems' capacities (3, 4). Interestingly, this novel coronavirus is able to be transmitted quite efficiently, affecting healthy adults and elderly people with higher rates of complications compared with other pandemics (5).

Evidence reported that COVID-19 represents a real threat for patients with comorbidities such as diabetes, hypertension, and cardiovascular, renal, or hepatic impairment (6, 7). Indeed, more severe cases with higher rates of mortality have been reported in older patients and in those with chronic illnesses such as cardiovascular disease. In this regard, patients affected from CKD (mainly those with DKD) are more likely to be affected since the rate of all-type infections and the presence of cardiovascular disease are greater than in the general population. The vulnerability of diabetic patients to be infected with different viruses has been reported. The evidence includes studies from the 2009 influenza A (H1N1) pandemic (8), SARS-CoV (9), and Middle East respiratory syndrome coronavirus (MERS-CoV) (10). Currently, the rapid spread of SARS-CoV-2 pandemic wait for new evidence in patients with DKD. However, as with many other conditions, marked alterations in the immune system have been reported in renal-affected patients. Beyond immune system impairment, special attention must be focused in the uremic state, excessive oxidative stress status due to retention of a plethora of toxins, and the accumulation of oxidative products that could worsen once the patient is infected.

It is known that SARS-CoV-2 targets respiratory cells; however, other organs might be affected for the invasion of the virus (namely the kidneys, ileum, and heart). A recent investigation identified that kidneys are organs with high a vulnerability to damage, according to angiotensin-converting enzyme 2 (ACE2) expression (11). Arterial smooth muscle and myocardial cells are also likely to be susceptible to SARS-CoV-2 damage (Figure 1). Of note, angiotensin-converting enzyme inhibitors (ACEi) do not inhibit ACE2 since ACE and ACE2 are different enzymes with two different active sites (12, 13). Moreover, although angiotensin II type 1 receptor blockers (ARB) are capable of upregulating ACE2 in experimental models, the evidence is not always consistent and differs among the diverse angiotensin II type 1 receptor blockers (12). Although the literature is controversial, the use of ACEi/ARB treatment does not affect the morbidity and mortality of COVID-19 combined with cardiovascular disease (14). To date, the actual evidence is unclear regarding a direct mechanism of kidney involvement in COVID-19. Nevertheless, mechanisms including a cytokine storm syndrome through sepsis pathways or direct viral renal tubular cells injury have been reported (15). At present, the main expression of renal damage in COVID-19 patients appear to be acute; however, some cases of macroalbuminuria/proteinuria and or haematuria may be associated with the endothelial dysfunction observed in these patients (Figure 1) (16).

**Figure 1:**
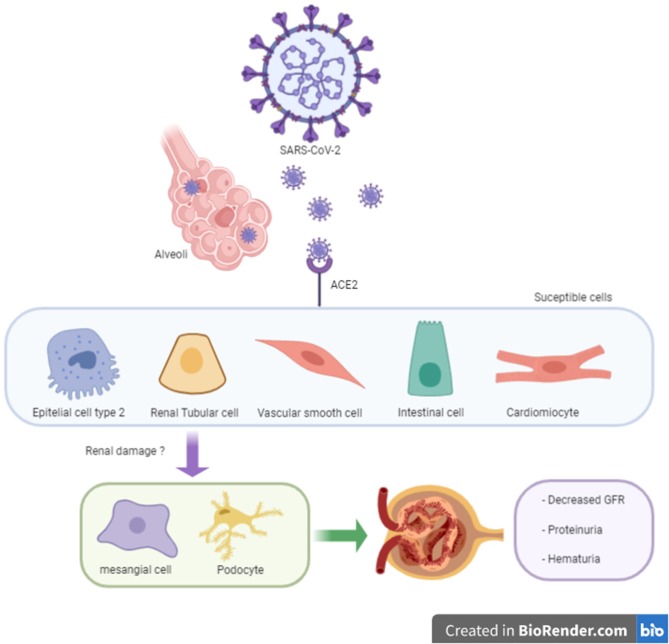
SARS-CoV-2 in susceptible cells expressing ACE2 and unknown renal damage.

What can we expect of these pandemics? As a merely hypothetical approach, we could observe the worsening of DKD, leading the patients to progress to a more severe stage of CKD or even to renal replacement therapies (RRT) or death. As we commented earlier, the actual evidence supports the notion that diminished immune defenses and other renal-related factors make diabetic patients more prone to certain infections. Finally, this pandemic will surely affect patients with renal-related illnesses more heavily, and mortality rates for these patients associated with the COVID-19 pandemic will require further research.

## Author Contributions

All authors listed have made a substantial, direct and intellectual contribution to the work, and approved it for publication.

## Conflict of Interest

JG has served as a consultant for Boëhringer-Ingelheim, Mundipharma, AstraZeneca, and Novonordisk and has received speaker honoraria from Boëhringer-Ingelheim, Mundipharma, AstraZeneca, Novonordisk, Novartis, and Eli Lilly. The remaining authors declare that the research was conducted in the absence of any commercial or financial relationships that could be construed as a potential conflict of interest.
